# Clinical Outcome of Ampullary Carcinoma: Single Cancer Center Experience

**DOI:** 10.1155/2019/3293509

**Published:** 2019-05-02

**Authors:** Mohammed Al-Jumayli, Amna Batool, Akshay Middiniti, Anwaar Saeed, Weijing Sun, Raed Al-Rajabi, Joaquina Baranda, Sean Kumer, Timothy Schmitt, Anusha Chidharla, Anup Kasi

**Affiliations:** Division of Medical Oncology, KU Cancer Center, The University of Kansas Health System, USA

## Abstract

**Introduction:**

Ampullary cancers represent a subset of periampullary cancers, comprising only 0.2% all gastrointestinal cancers. Localized disease is primarily managed by a surgical intervention, called pancreaticoduodenectomy (PD), followed in many cases by the administration of adjuvant chemotherapy (CT) or chemoradiation therapy (CRT). However, there are no clear evidence-based guidelines to aid in selecting both the modality and regimen of adjuvant therapy for resected Ampullary carcinoma.

**Methods:**

We retrospectively analyzed 54 patients at KU Cancer Center, who had undergone endoscopic resection or pancreaticoduodenectomy (PD) for Ampullary cancer from June 2006 to July 2016. We obtained patients' baseline characteristics, clinical presentation, pathology, treatment modality, recurrence pattern, and survival outcomes. The time-to-events data were compared using Kaplan-Meier methods. A univariate and multivariate Cox proportional hazards regression was performed to evaluate factors associated with overall survival (OS) and generate hazard ratios (HR).

**Results:**

The mean age of the 54 patients was 68 (37-90). 38 (70%) were males and 16 (30%) were females. Most of the patients were Caucasian (76%). Approximately half of all patients had a history of smoking, 20% had alcohol abuse, and 13% had pancreatitis. Among the 54 patients with localized cancers, 9 (16%) were treated definitively with nonoperative therapies, usually due to a prohibitive comorbidity profile, performance status, or unresectable tumor. 45 out of 54 patients (83%) underwent surgery. Of the 45 patients who underwent surgery, 18 patients (40% of the study cohort) received adjuvant therapy due to concerns for advanced disease as determined by the treating physician. 13 patients (24%) received adjuvant CT and 5 patients (9.2%) received CRT. The remaining 27 patients (50%) underwent surgery alone. The median OS for the entire study cohort was 30 months. When compared to surgery alone, adjuvant therapy with either CT or CRT had no statistically significant difference in terms of progression-free survival (*p*=0.56) or overall survival (*p*=0.80). In univariate Cox proportional hazards regression analysis, high-risk features like peripancreatic extension (16%) and perineural invasion (26%) were found to be associated with poor OS. Lymph node metastasis (29%) did not significantly affect OS (HR 1.42, 95% CI [0.73-1.86];* p*=0.84). Lymphovascular invasion (29%) was not associated with poor OS (HR 1.22, 95% CI [0.52, 2.96];* p*=0.76). In multivariate Cox regression analysis, only age group>70 years was significantly associated with OS , while other factors, including the receipt of adjuvant therapy, lymph nodes, positive margin, and lymphovascular, perineural, and peripancreatic involvement, were not significantly associated with OS. These results are likely due to small sample size.

**Conclusions:**

Despite numerous advances in both cancer care and research, efforts in rare malignancies such as Ampullary cancer remain very challenging with a clear lack of an evidence-based standard of care treatment paradigm. Although adding adjuvant therapies such as chemotherapy or chemoradiotherapy is likely to improve survival in high-risk disease, there is no standardized regimen for the treatment of Ampullary cancer. More research is required to elucidate whether statistically and clinically relevant differences exist that may warrant a change in the current adjuvant treatment strategies.

## 1. Introduction

Ampullary carcinoma is a rare malignant tumor originating from the Ampulla of Vater [1, 2] The reported incidence is less than one per 100,000 in an autopsy series and in the general population comprises of 0.2% of all gastrointestinal tumors [1]. In 90% of patient cases, this represents a primary presentation with a predominance of Caucasian males being affected over other races and gender [3]. In patients with hereditary polyposis syndromes, the incidence of Ampullary cancer is multifold and presents at an earlier age warranting surveillance endoscopy [4].

Previous retrospective studies have assessed the difference in prognosis of Ampullary carcinomas based on immunohistologic subtypes. Pancreaticobiliary subtype versus intestinal subtype showed a significant difference of almost 10-fold in median survival, 16 vs. 116 months, respectively [5]. In addition to immunohistochemical subtypes, nodal status was also found to have an impact on survival. Patients with node-negative, nonpancreatobiliary type had an excellent prognosis with 5-year survival rate of 88%, while those with node-positive, pancreatobiliary type had a poor prognosis with a 5-year survival of 20%. Patients with node-positive, nonpancreaticobiliary or node-negative, pancreatobiliary type had an intermediate prognosis with a 5-year survival of 47%. A recent meta-analysis reported that pancreatobiliary type predicted a worse overall survival (hazard ratio [HR] 1.84, 95% CI 1.49- 2.27;* p*< 0.001) and disease-free survival (HR 1.93, 95% CI 1.23-3.01;* p*=0.004) [6]. On the other hand, other studies failed to replicate similar findings, limiting their utility in diagnostic algorithms. Emerging evidence suggests an increased complexity of tumor subtypes such as the existence of a mixed subtypes which warrants further research instead of making treatment decisions based on immunohistologic subtypes alone [7].

Patients with localized disease are primarily managed by pancreaticoduodenectomy (PD), often followed by the administration of adjuvant chemotherapy (CT) or chemoradiation therapy (CRT) [2]. However, given the paucity of clinical evidence, current treatment recommendations in the adjuvant setting are not included in published consensus like NCCN or ESMO guidelines [8]. Retrospective and prospective studies have investigated the role of adjuvant therapy in this context; however, the evidence remains inconclusive. Our retrospective, single-center study was conducted to evaluate whether any survival advantages exist with either adjuvant chemotherapy or chemoradiotherapy compared to surgery alone in the management of resected Ampullary carcinoma.

## 2. Methods

This was a retrospective study conducted at the Kansas University Cancer Center. Data was collected on patients with a diagnosis of Ampullary adenocarcinoma who were treated at our institution between 2006 and 2016. Our study enrollment was over a period of 10 years from 2006 to 2016 with a followup of at least 2 years for each patient in order to obtain data relevant for long-term survival analysis.

The Institutional Review Board approved this retrospective study. Data collection on eligible patients included patient baseline characteristics, clinical presentation, pathology, treatment modality, recurrence, and survival. Statistical analysis was performed using SPSS 22.0 with statistical significance established at* p*<0.05. Both the Kaplan-Meier method and log-rank were used to compare the time-to events. Univariate and multivariate Cox proportional hazards regression model were created to identify factors associated with OS. Of note, many of the older cases included in the study had no immunostaining performed by pathologists. Hence, we were unable to evaluate prognostic association with immunohistologic subtypes.

## 3. Results

A total of 54 patients with Ampullary adenocarcinoma were selected for evaluation. The epidemiology, clinical presentation, pathologic features, and staging of Ampullary carcinoma at baseline are presented in Table 1. The mean age of our patient cohort was 68 years. There were more males patients than females, 38 vs. 16 respectively. Most of our patients were Caucasians (76%). Approximately half of the patients (52%) had a history of smoking, 20% were alcohol abusers, and 13% had a history of pancreatitis.

**Figure 1:**
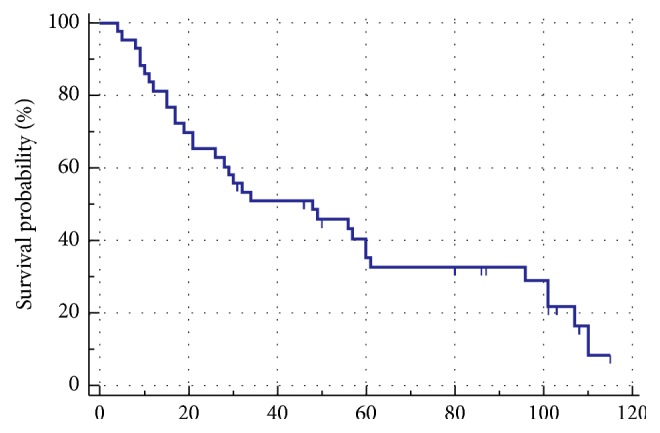
Overall survival (OS) of study cohort.

**Figure 2:**
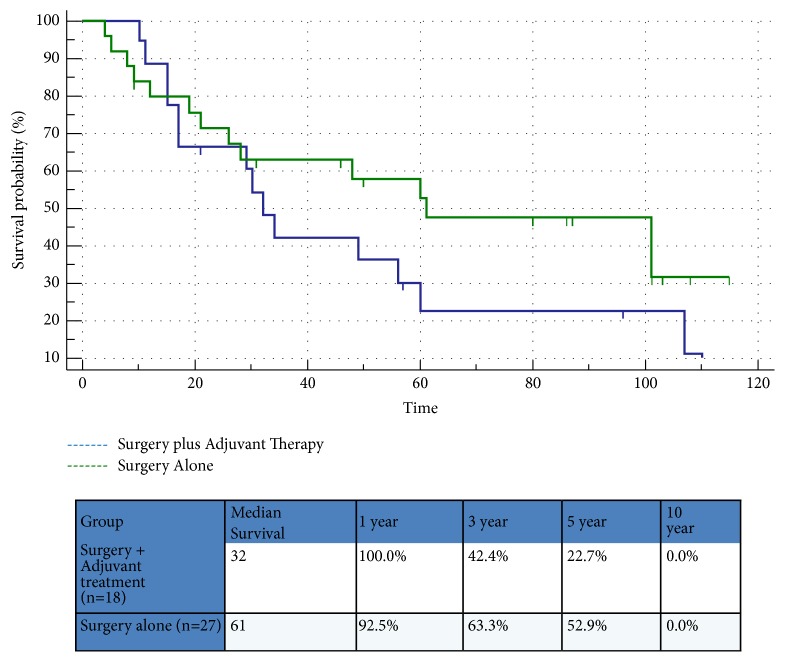
Survival following surgery plus adjuvant therapy vs. surgery alone.

**Figure 3:**
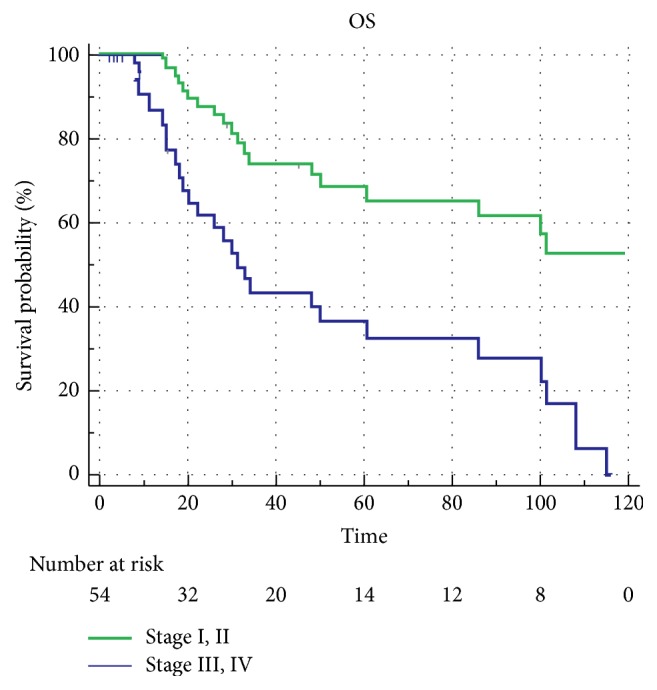
OS probability according to TNM staging.

Nine patients were treated nonoperatively, secondary to a prohibitive comorbidity profile, performance status, or an unresectable tumor. Adjuvant treatment was administered after surgery because of concerns for advanced disease determined by the treating physician in 18 patients: 13 (24%) received chemotherapy and 5 (9.2%) received chemoradiation therapy. The remaining 27 (50%) patients underwent surgery alone. The median OS for the study cohort was found to be 30 months (Figure 1). Recurrence was noted in 40% of patients who underwent surgery. Patients treated with adjuvant therapies following PD had a more locally advanced disease than those who had surgery alone. Most patients who received adjuvant therapy after surgical resection had positive prognostic features (50% had positive LN and 40% had T4 disease). When compared to surgery alone, adjuvant chemotherapy or chemoradiation therapy failed to demonstrate a statistically significant difference in terms of progression-free survival (PFS) (*p*=0.56) or OS (*p*=0.80) (Figure 2). When patients with TNM stage I or II disease were compared to patients with TNM stage III or IV disease, a median survival difference was noted; however, this difference was not at the statistical significance level (*p*=0.7) due to the small number of patients in stage I and II cohort (Figure 3). The 5-year survival rates of patients who underwent surgery alone in our cohort were 53%, which is within the 30-60% historical range published in the literature.

On Univariate Cox proportional hazards regression, peripancreatic extension (16%) and perineural invasion (26%) were found to be associated with poor OS (Table 2). Lymph node metastases were present in 29% of the cohort but did not significantly affect OS (HR 1.42, 95% CI [0.73- 1.86];* p*=0.84). Lymphovascular invasion was present in 29% but did not affect OS (HR 1.22, 95% CI [0.52, 2.96];* p*=0.76).

In the multivariate Cox regression analysis, only age group >70 years was significantly associated with OS. The other general and local prognostic factors including the receipt of adjuvant therapy (chemotherapy/radiation), lymph nodes, positive margin status, and lymphovascular, perineural, and peripancreatic extension were not significantly associated with OS (Table 3). These results were largely attributed to the small sample size.

## 4. Discussion

Ampullary cancer has a better prognosis when compared to pancreatic cancer or cholangiocarcinoma, largely in part due to the location of the tumor which is associated with an early onset of biliary obstruction-associated jaundice and thus early disease detection [2, 9]. Since half of all Ampullary carcinomas are anticipated to recur following the initial intervention, it is of paramount importance that features associated with recurrence risk are identified and managed accordingly [10]. This risk is highlighted by the fact that up to 28% of patients with T1 disease have been reported having lymph node metastases [11]. This is the primary reason why PD is preferred over local Ampullectomy as determining benign vs. malignant tumor status is not routinely feasible using only preoperative symptoms or lesion size as predictors. Due to their earlier presentation, resection remains the only curative treatment for patients with Ampullary cancer and is feasible in approximately 50% compared to that of less than 10% in pancreatic adenocarcinoma. The mismatch between tumor size and biliary obstruction explains why, compared to pancreatic cancers, resectability of Ampullary cancer at presentation is significantly higher. As a result, the prognosis is considerably better than that for pancreatic cancer [11] However, despite such an aggressive surgical intervention, most patients will have a disease recurrence, hence justifying a possible role for adjuvant therapies.

The role of postsurgical adjuvant treatment of Ampullary cancer remains to be established, because of limited data available in this rare disease. Preoperative neoadjuvant radiation, chemotherapy, or chemoradiation is the available options and has been studied with a survival benefit evident in certain subset populations: patients with multiple morbidities who need preoperative optimization which may delay surgery; patients with poor biologic behavior of neoplasm; patients with the possibility of an interruption in therapy due to postoperative surgical complications [12]; or advanced disease with poor prognostic features. A significant proportion of our cohort consisted of patients with advanced disease as indicated by perineural invasion rate (26%), extension into adjacent organs (37%), and peripancreatic soft tissue (16%).

Our study failed to demonstrate a survival benefit with postoperative adjuvant therapy when compared to those who had no adjuvant therapy. This is likely due to the small number of patients available for comparison at our institution. It is reasonable to assume that patients treated with adjuvant therapies following PD had a more advanced disease than those who had surgery alone. In our study, for example, most patients who received adjuvant therapy after surgical resection had positive prognostic features (50% had positive LN and 40% had T4 disease). The 5-year survival rates of patients who underwent surgery alone in our cohort were 53%, which is within the 30-60% historical range published in the literature albeit towards the upper part of the range [13, 14]. This may be due to the use of a standardized dissection at the time of PD [15]. To the best of our knowledge, resection decisions were not based in part upon histological subtypes. The limitations of our study include a single institution analysis, retrospective design, and a small sample size that prohibited analyses with adequate power. Another possible limitation of our study is that, given the small sample size, we did not control for chemotherapy regimen or were able to compare the efficacy of combination over single-agent chemotherapy, as it was previously reported [16]

The poor OS observed in our study was likely due to the high rate of tumor invasion and extension. High-risk features such as lymph node metastases, lymphovascular invasion, peripancreatic extension, and perineural invasion have been known to impact OS [17]. Perineural invasion is a predictive factor for lymph node metastases: odds ratio (OR) of 3.0 [11]. Other predictive factors of lymph node metastases include tumor size ≥1 cm (OR 2.1), poor histological grade (OR 4.8), microscopic vessel invasion (OR 6.6), and depth of invasion > pT1 (OR 4.3; all* p*< 0.05). In a recent study by Zhao and colleagues, the degree of tumor infiltration correlated with recurrence (*p*=0.014). Extraduodenal local resection (*p*=0.026) was associated with increased survival [9]. Given the significant association of survival reported with peripancreatic and perineural tumor extension, which was also found in our study, this indicates a role for adjuvant therapy especially in high-risk disease. In accordance to survival trends published in literature, patients with either stage I or II Ampullary cancer demonstrated a trend toward survival benefit over more advanced stages [9].

The role of adjuvant postoperative chemotherapy or chemoradiation for Ampullary Adenocarcinoma has been investigated previously in a few studies with conflicting results. The ESPAC-3 trial randomized patients with periampullary cancers (69.4% [n=297] with Ampullary adenocarcinoma) who underwent PD to either observation or adjuvant chemotherapy. Although no significant differences were seen in the primary analysis, adjuvant chemotherapy with fluorouracil plus folinic acid or gemcitabine was associated with improved survival after controlling for prognostic variables [16]. However, both the multicentered EORTC-40891 trial in patients with periampullary cancers (44% [n=92] had Ampullary cancer) and a single-center study in India (n=104) failed to show a survival benefit with adjuvant chemoradiation (with fluorouracil) following PD [18, 19]. Both studies were powered adequately to detect significant differences.

On the other hand, at least one recent meta-analysis (n=3361 patients) using a pooled analysis reported a significant survival benefit of adjuvant chemoradiation (HR=0.75,* p*=0.01) following PD [20]. Furthermore, they also found strong associations between postoperative chemoradiation and survival in patients with positive lymph nodes and T3/T4 tumors, although they had a limited number of eligible subjects (n=3) for this subgroup analysis [20].

Retrospective studies have supported the selective administration of adjuvant or neoadjuvant therapy to patients with periampullary cancers with high-risk features. Bhatia et al. found a significant survival benefit of adjuvant chemoradiation for patients with lymph node positive Ampullary cancer (23% [n=29]) [17]. Other retrospective studies including Lee et al. (33% [n=13] received adjuvant chemoradiation) and Narang et al. (55% [n=66] received adjuvant chemoradiation) found a survival benefit albeit only after multivariate analysis [13, 21]. Interestingly, most recently a retrospective analysis of patients from the Surveillance, Epidemiology, and End Results (SEER) database found significantly longer cancer-specific survival and OS in patients with N2 nodal status who underwent adjuvant radiotherapy without concomitant chemotherapy [22].

Most relevant in comparison to our findings were results from a similar single-center study consisting of 52 patients who underwent potentially curative PD [23]. As in our study, no survival benefit was observed with adjuvant therapy; however, they did observe a trend towards improved survival with chemoradiation as opposed to chemotherapy. Perineural invasion was associated with decreased survival, as observed in our study. Lymphovascular invasion was associated with decreased survival, unlike our study. These differences between study findings are likely due to the small patient population available for analysis yet are relevant findings for prognostication. Findings from our single-center study along with evidence from other studies support the rationale for more prospective investigations to establish the role of adjuvant therapies in the postsurgical setting especially in higher-risk patients with Ampullary adenocarcinoma.

Ampullary adenocarcinoma is a rare cancer and most series have relatively small numbers. As a result, analysis of factors influencing outcome has been limited. The strengths of the current study include its comprehensive study of the prognostic factors of such a rare cancer extracted from a prospectively maintained database with robust clinical data. However, several limitations should be acknowledged. First, this analysis suffers from the limitations inherent in its retrospective, single-institution design. Second, the surgical resectability criteria for patients who underwent surgery and those who did not are difficult to determine retrospectively and therefore introduce physician/patient bias that is not uncommon in a retrospective study. Third, the adjuvant chemotherapy regimens used may have had variable effects; however, no standardized regimen in adjuvant setting exists for Ampullary cancer. Finally, there was no stratification by histological subtype and/or other molecular profiling due to unavailability of archival tissue. The multivariate analysis of prognostic features did not reach statistical significance due to small sample size in our study. Hence, in future, a multicenter prospective study with a larger sample size is warranted to conduct analyses with adequate power.

## 5. Conclusions

Despite numerous advances in cancer care and research, efforts in rare malignancies such as Ampullary cancer remain very challenging with a clear lack of an evidence-based standard of care treatment paradigm. Although adding adjuvant therapies such as CT or CRT likely improves survival in patients with high-risk disease, no standardized regimen exists for the treatment of Ampullary cancer. More research is required to elucidate whether clinically relevant differences exist that may warrant a change in existing treatment strategies. Stratification by histological subtype, staging, prognostic factors, and/or other molecular profiling in large studies may enable personalized treatment decision making.

## Figures and Tables

**Table 1:** Baseline Characteristics.

Variable	Results (*n*=54) Mean (%)
Age (years)	
n	54
Mean	68
STD	11.2

Gender	
Female	16(30%)
Male	38(70%)

Race	
Caucasian	41 (76%)
African American	3 (5%)
Other	10(19%)

Comorbidities	
Smoking	28(52%)
Alcohol Use	11(20%)
History of Pancreatitis	7(13%)
Diabetes	19(35%)

Tumor Characteristics	
ECOG Score 0-1	15(28%)
Elevated CA-19 (>34)	19(36%)
Biliary Stenting (pre-OP)	39(74%)
Tumor Histology	28(44%)
Ductal Adenocarcinoma	38(72%)
Adenocarcinoma not otherwise specified	8(15%)
Mucinous Adenocarcinoma	4(9%)

Tumor Grade	
G1: Well Differentiated	5(9%)
G3: Moderately Differentiated	33(61%)
Unknown	16(29%)

Tumor stages(TNM)	
Stage I	13(24%)
Stage II	9(20%)
Stage III	25(44%)
Stage IV	7(12%)

**Table 2:** Univariate Analyses.

Characteristics	Hazard Ratio (95% CI)	P-value
Age	1.22 (0.52-2.96)	*0.6291*

Gender (Female vs. Male)	1.06 (0.70-1.06)	*0.8504*

Smoking (Yes vs. No)	1.24 (0.48-1.78)	*0.7920*

Diabetes (Yes vs. No)	0.91 (0.42-1.02)	*0.5930*

Lymphovascular	1.22 (0.52-2.96)	*0.769*

invasion (Yes vs. No)		

Lymph node status (+ve vs. -ve)	1.42 (0.73-1.86)	*0.843*

Perinodal invasion	0.67 (0.49-3.21)	*0.709*

(Yes vs. No)		

Peripancreatic	1.78 (1.42-2.65)	*0.0459*

extension (Yes vs. No)		

Perineural extension (Yes vs. No)	*1.62 (1.33-3.02)*	*0.0352*

**Table 3:** Multivariate Cox proportional hazards regression for overall survival.

characteristics	Hazard Ratio (95% CI)	p- Value
*Age*		

60-70	1.56 (0.49–4.9)	0.45

>70	3.18 (1.0–10.13)	0.04

*Albumin ( >3.5 vs. < 3.5)*	1.19 (0.66–2.13)	0.56

Margin(+ve vs. -ve)	1.06 (0.26-4.63)	0.193

*Lvi (yes vs. no)*	1.68 (0.55–5.12)	0.36

*Ln (+ vs. -)*	1.78 (0.35- 1.32)	0.1382

*Pni (yes vs. no)*	1.83 (0.62–5.38)	0.6276

*Peripancreatic (Y vs. No)*	*4.55 (1.94, 10.64)*	0.783

*P-stage (>2 vs. <=2)*	1.46 (0.46–4.77)	0.53

*Adjuvant Therapy*	1.14 (0.56–2.31)	0.72

## Data Availability

Deidentified data may be provided to researchers upon approval by the University of Kansas Health System after an agreement for data sharing is executed.
